# Supplementary material for: Efficient Generation and Correction of Mutations in Human iPS Cells Utilizing mRNAs of CRISPR Base Editors and Prime Editors
Source: Genes (Basel). 2020 May 6;11(5):511. doi: 10.3390/genes11050511 (PMC7288465; doi:10.3390/genes11050511)
Supplement: Supplementary file 1 [file genes-11-00511-s001.pdf]

genes

MDPI

Supplementary Files

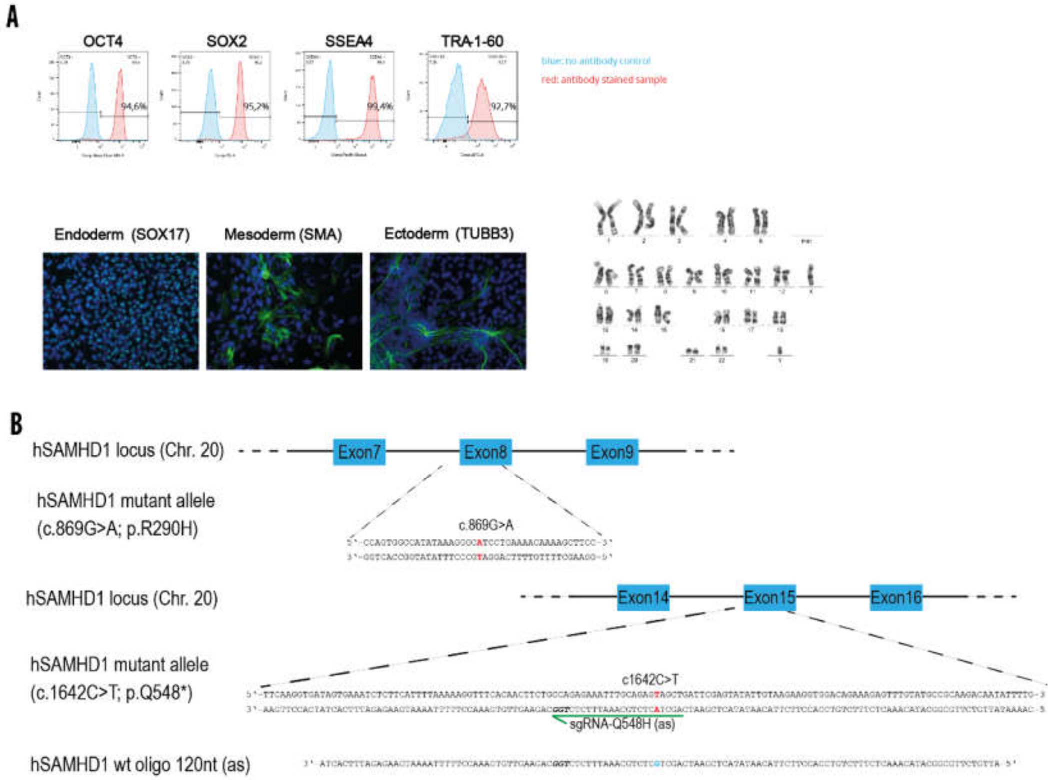

C

Sequencing result	dead clones	Indels	unedited	mixed clones	repaired
HDR	2/72	2/72	68/72	0/72	0/72
	2.8%	2.8%	94.4%	0%	0%

Figure S1. HDR-based repair strategy of a SAMHD1 mutation. (**A**) Validation of hiPSCs derived from a patient. The expression of indicated pluripotency markers of the patient SAMHD1 hiPSC were investigated by flow cytometry and via three-germlayer-differentiation (SOX17, SMA, and TUBB3) as well as karyotyping by G-banding. (**B**) Schematic presentation of mutated SAMHD1 alleles and the genome editing strategy to correct the exon 15 mutation by HDR. The sgRNA-targeting sequence is shown by an arrow and the protospacer-adjacent motif (PAM) sequence is indicated in bold italicized. The nonsense mutation (c.1642C>T) is in red. Below the 120 nt long ssDNA oligonucleotide HDR donor to correct the mutation with the correcting nucleotide shown in blue. (**C**) Editing rate of the *SAMHD1* gene. Absolute numbers and percentages of investigated clones are provided. Almost 95% of the clones are unedited and two clones show indels.

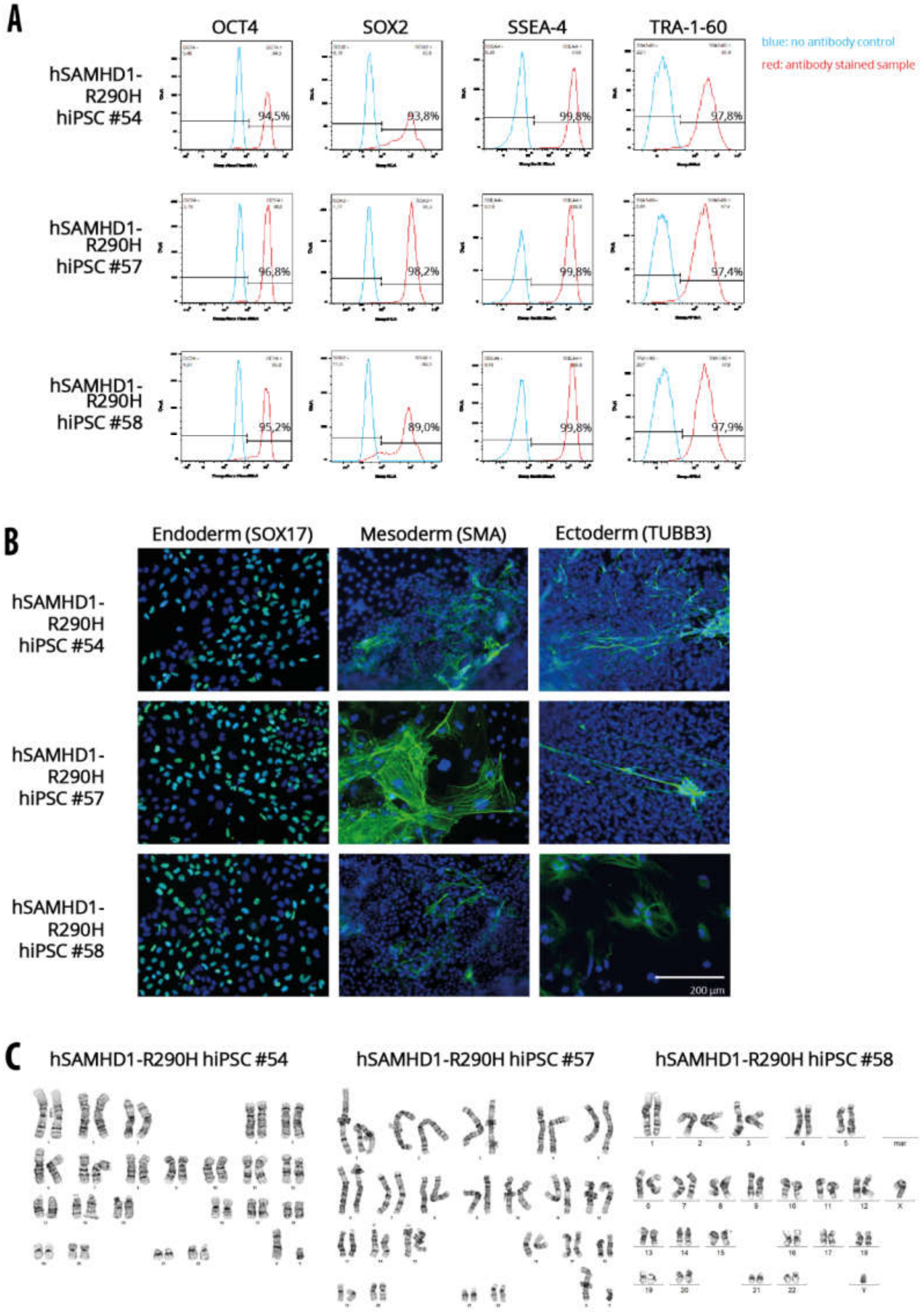

Figure S2. Validation of three SAMHD1 corrected hiPSC clones. (**A**) The expression of indicated pluripotency markers of clones were investigated by flow cytometry. (**B**) Fluorescent images showing differentiation to all three germlayers. Investigated markers and the three subclones (54, 57, and 58) studied are shown. (**C**) Chromosome spreads of indicated iPSC subclones revealed a normal karyotype (46, XY).

genes

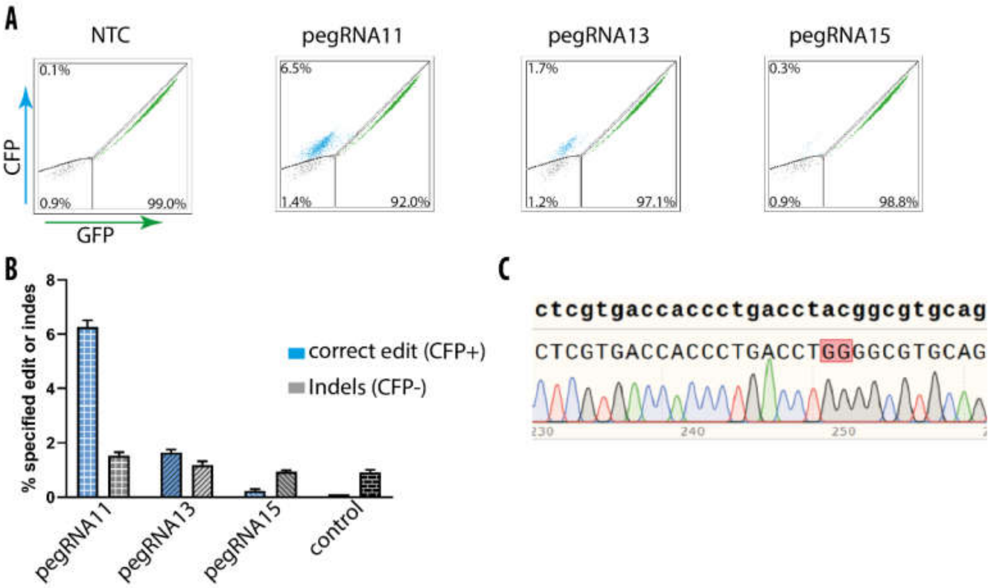

Figure S3. Conversion of GFP-to-CFP in HEK293T cells with PE. (**A**) FACS profiles of HEK293T cells 14 days post transfection of the PE. Percentages of the gated fractions are shown. (**B**) Quantification of editing efficiencies based on FACS results. Results are represented as mean ± SEM of three independent experiments. (**C**) Sanger sequencing data from CFP positive sorted cells. Converted nucleotides are shown in a red box.

genes

MDPI

Table S1. Tested conditions for mRNA delivery into hiPS cells.

hiPSC line	medium	coating/plate	passaging agent	mRNA (amount)	transfection reagent (amount)	transfection efficiency
AAVS1-eGFP	mTeSR1	VN	TrypLE	mCherry (4.7 pmol)	TransIT-LT1 (4.5 µl)	28.4%
	mTeSR1	VN	ReLeSR (sc)			41.2%
	Essential 8 Flex	VN	Accutase			0.5%
	Essential 8 Flex	VN	ReLeSR (sc)			1.1%
	Essential 8 Flex	VN->FN1	Accutase			1.0%
	Essential 8 Flex	VN->FN1	ReLeSR (sc)			1.2%
	Essential 8 Flex	FN1	Accutase			0.0%
	Essential 8 Flex	FN1	ReLeSR (sc)			0.2%
AAVS1-eGFP	Essential 8 Flex -> 48 h mTeSR1	VN	ReLeSR (sc)	mCherry (5 pmol)	TransIT-LT1 (2 µl)	7.2%
					TransIT-LT1 (4 µl)	11.4%
					TransIT-LT1 (6 µl)	13.1%
					TransIT-LT1 (8 µl)	14.5%
					TransIT-LT1 (10 µl)	18.2%
					TransIT-LT1 (12 µl)	25.1%
AAVS1-eGFP	Essential 8 Flex -> 48 h mTeSR1	VN	ReLeSR (sc)	mCherry (5 pmol)	TransIT-LT1 (5 µl)	16.7%
				mCherry (7.5 pmol)	TransIT-LT1 (7,5 µl)	15.2%
				mCherry (10 pmol)	TransIT-LT1 (10 µl)	26.5%
				mCherry (20 pmol)	TransIT-LT1 (20 µl)	15.5%
				mCherry (30 pmol)	TransIT-LT1 (30 µl)	18.1%
				mCherry (50 pmol)	TransIT-LT1 (50 µl)	20.9%
CRTD2	mTeSR1	Matrigel	TrypLE	eGFP (3 pmol)	nucleofection (100 µl reaction)	86.6%
				eGFP (7.5 pmol)	nucleofection (100 µl reaction)	92.3%
				eGFP (10 pmol)	TransIT-LT1 (10 µl)	0.1%
CRTD3	mTeSR1	Matrigel	TrypLE	eGFP (3 pmol)	nucleofection (100 µl reaction)	87.4%
				eGFP (7.5 pmol)	nucleofection (100 µl reaction)	93.0%
				eGFP (10 pmol)	TransIT-LT1 (10 µl)	0.1%
F8	DEF-CS	VN	Accutase	eGFP (8 pmol)	TransIT-LT1 (12 µl)	56.9%
				eGFP (12 pmol)	TransIT-LT1 (12 µl)	70.4%
				eGFP (16 pmol)	TransIT-LT1 (12 µl)	84.3%
				eGFP (10 pmol)	Lipofectamin messenger max (4 µl)	95.9%

genes

MDPI

F8	DEF-CS	VN	TrypLE	TagBFP (20 pmol)	Lipofectamin messenger max (3.75 µl)	91.2%

genes

Table S2. Editing rate of the *SAMHD1* gene plasmid vs. mRNA.

**Sequencing result**	**dead clones**	**Indels**	**unedited**	**mixed clones**	**repaired**
ABE-Plasmid	10/48	0/48	21/48	17/48	0/48
	20.3%	0%	43.8%	35.4%	0%
ABE-mRNA	5/21	0/21	6/21	6/21	4/21
	23.8%	0%	28.6%	28.6%	**19.0%**

genes

MDPI

Table S3. BE-mediated mRNA editing of *TP53* in hiPS cells. Important features of the selected TP53 mutations are presented. The introduced mutations; frequencies of the mutations in the cosmic database; the sequence of the employed sgRNAs; the position of the edited nucleotide in the protospacer, which BE was used for which modification; and the correct editing rate and the percentage of unexpected modifications are shown.

**Protein**	**cDNA**	**Frequency in cosmic**	**sgRNA**	**Position (PAM distal start)**	**BE**	**Expected modifications (%)**	**No expected modifications**
p.C141R	c.421T>C	9x	ccaACTGGCCAAGACC**T**GCCCTG	7	ABE	52.4%	none detected
p.Y163H	c.487T>C	11x	ccgCGCCATGGCCATC**T**ACAAGC	7	ABE	86.3%	none detected
p.H193R	c.578A>G	75x	CAGC**A**TCTTATCCGAGTGGAagg	5	ABE	27.0%	none detected
p.C135Y	c.404G>A	37x	cctCAACAAGATGTTTT**G**CCAAC	6	CBE	6.4%	4.7%
p.C141Y	c.422G>A	44x	ccaACTGGCCAAGACCT**G**CCCTG	6	CBE	8.8%	3.3%
p.C238Y	c.713G>A	74	ccaCTACAACTACATGT**G**TAACA	6	CBE	35.4%	15.4%
p.R273C	c.817C>T	423x	GTG**C**GTGTTTGTGCCTGTCCtgg	4	CBE	<1%	11.3%
p.Q165*	c.493C>T	28x	CAAG**C**AGTCACAGCACATGAcgg	5	CBE	2.5%	1.0%
p.R213*	c.637C>T	314x	CACTTTT**C**GACATAGTGTGGtgg	8	CBE	18.3%	1.9%

Table S4: Oligo nucleotides used in this study.

**Oligo's and sgRNA**	**Sequence (5′-3′)**
**hSAMHD1 PCR and sequencing primers**	
SAM-15b fw	CTCCAATGTGTGACTTCAAGGTG
SAM-15b rev	CCCAACTCCTGTAGGAAGAAATC
**EGFP PCR and sequencing primers**	
SFFVp fw	GAGCTCACAACCCCTCACTC
WPRE rev	AGCAACATAGTTAAGAATACCAGTC
***TP53* PCR and sequencing primers**	
H193R fw	ACACTCTTTCCCTACACGACGCTCTTCCGATCTCAGGCCTCTGATTCCTCACT
H193R rev	GTGACTGGAGTTCAGACGTGTGCTCTTCCGATCTCCAGTTGCAAACCAGACCTC
C141R-C141Y-C135Y fw	ACACTCTTTCCCTACACGACGCTCTTCCGATCTAGTACTCCCCTGCCCTCAAC
C141R-C141Y-C135Y rev	GTGACTGGAGTTCAGACGTGTGCTCTTCCGATCTGCCTCACAACCTCCGTCAT
Y163H-Q165* fw	GTGACTGGAGTTCAGACGTGTGCTCTTCCGATCTAGTACTCCCCTGCCCTCAAC
Y163H-Q165* rev	ACACTCTTTCCCTACACGACGCTCTTCCGATCTGCCTCACAACCTCCGTCAT
R213* fw	GTGACTGGAGTTCAGACGTGTGCTCTTCCGATCTCAGGCCTCTGATTCCTCACT
R213* rev	ACACTCTTTCCCTACACGACGCTCTTCCGATCTCCAGTTGCAAACCAGACCTC
C238Y fw	GTGACTGGAGTTCAGACGTGTGCTCTTCCGATCTCTTGGGCCTGTGTTATCTCC
C238Y rev	ACACTCTTTCCCTACACGACGCTCTTCCGATCTCCAGTGTGATGATGGTGAGG
R273C fw	GTGACTGGAGTTCAGACGTGTGCTCTTCCGATCTGCCTCTTGCTTCTCTTTTCC
R273C rev	ACACTCTTTCCCTACACGACGCTCTTCCGATCTTTGCGGAGATTCTCTTCCTC
**HDR template oligos**	
hSAMHD1 wt oligo (antisense)	ATTGTCTTGCGGCATACAAACTCTTTCTGTCCACCTTCTTACAATATACTCGAATCAGCT**G**CTCTGCAAATTTCTCTGGCAGAAGTTGTGAAACCTTTTTAAAATGAAGAGATTTCACTA
**IVT mRNA production primers**	
EGFP-5′	GCTAATACGACTCACTATAGGGAGAGCCGCCACCATGCCAAAAAAGAAGAG

EGFP-3'	TTTTTTTTTTTTTTTTGGTTTATTCCTAGTACAGCTCGTCCATGCC
mCherry-5'	GCTAATACGACTCACTATAGGGAGAGATGGTGAGCAAGGGCGAG
mCherry-3'	TTTTTTTTTTTTTTTTGGTTTATTCTTACTTGTACAGCTCGTCCATGC
tagBFP-5'	GCTAATACGACTCACTATAGGGAGAGATGAGCGAGCTGATTAAGGAGA
tagBFP-3'	TTTTTTTTTTTTTTTTGGTTTATTCTTAATTAAGCTTGTGCCCCAGTT
ABE-5'	GCTAATACGACTCACTATAGGGAGAGCCGCCACCATGGATTACAAAG
ABE-3′	TTTTTTTTTTTTTTTTGGTTTATTCTATTTCTTTTTCTTAGCTTGACCAG
ABE_Puro-3′	TTTTTTTTTTTTTTTTGGTTTATTCTATCAGGCACCGGGCTTGCGGG
CBE-5′	GCTAATACGACTCACTATAGGGAGACCGCCACCATGGACTATAAGG
PE2-5′	GCTAATACGACTCACTATAGGGAGAGCCACCATGAAACGGACAGCCG
PE2-3′	TTTTTTTTTTTTTTTTGGTTTATTCTTAGACTTTCCTCTTCTTCTTGGGC
